# Pharmakotherapie und intensivmedizinische Aspekte des Status epilepticus: Update 2020/2021

**DOI:** 10.1007/s00101-021-01000-y

**Published:** 2021-07-01

**Authors:** Gabrielė Saitov, Annekatrin Müller, Börge Bastian, Dominik Michalski

**Affiliations:** 1 Klinik und Poliklinik für Anästhesiologie und Intensivtherapie, Universitätsklinikum Leipzig, Liebigstr. 20, 04103 Leipzig, Deutschland; 2 Klinik und Poliklinik für Neurologie, Universitätsklinikum Leipzig, Liebigstr. 20, 04103 Leipzig, Deutschland

**Keywords:** Epileptische Anfälle, Konvulsiver Status, Nonkonvulsiver Status, Intensivtherapie, Stufentherapie, Seizure emergencies, Non-convulsive status, Convulsive status, Intensive care, Stage-based treatment

## Abstract

Die gezielte Therapie epileptischer Ereignisse und im Speziellen des Status epilepticus (SE) setzt das sichere Erkennen der Krankheitsbilder voraus, wofür gerade bei Formen mit vorwiegend nichtmotorischen Symptomen klinische und elektroenzephalographische Expertise notwendig ist. Die im Jahr 2020 erfolgte Fortschreibung der deutschen Leitlinie zur Behandlung des SE hält an der streng stufengerechten Therapie fest, die eskalierend die Anwendung von Benzodiazepinen, spezifischen Antiepileptika und Anästhetika vorsieht. Bisher ist die Eingrenzung eines in den allermeisten Fällen wirksamen sowie zugleich sicheren und interaktionsfreien Antiepileptikums nicht gelungen. Individuelle Vorerkrankungen und aktuelle Begleitumstände gehen daher genauso wie Erfahrungen des Behandlerteams in die differenzierte Behandlung des SE ein. Insbesondere bei therapierefraktären Formen des SE erweist sich die Therapie als durchaus kompliziert und hat regelhaft intensivmedizinische Implikationen. Mithin ergeben sich im Zuge der modernen SE-Behandlung zahlreiche interdisziplinäre Schnittstellen. Zukünftige wissenschaftliche Fragstellungen werden sich u. a. mit der optimalen Therapie des nonkonvulsiven SE und hier v. a. dem Ausmaß und dem Zeitpunkt von adäquaten Therapieschritten sowie mit assoziierten ethischen Fragen einer Therapieeskalation beschäftigen.

## Abstract (en)

Focused treatment of epileptic emergencies, and in particular status epilepticus (SE), require a reliable differentiation of epileptic syndromes. In these cases, and especially in cases with predominant non-motor symptoms, clinical and electroencephalographic expertise is necessary. In 2020 the German guidelines for the management of SE were updated, which adhere to a strict stage-based treatment algorithm. The staged approach includes the administration of benzodiazepines, antiepileptic drugs and anesthetic agents. So far, efforts failed to determine the most effective and safest antiepileptic drug without interaction potential. Therefore, for the differentiated treatment of SE, individual pre-existing medical conditions and concomitant circumstances must be considered, added by the experience of the medical team. Therapeutic interventions especially for refractory forms of SE have been shown to be complex with relevant implications concerning intensive care aspects. Consequently, the modern treatment strategy of SE is characterized by an interdisciplinary approach. Future research is needed to define the optimal treatment of non-convulsive SE, in particular regarding the time point and degree of treatment escalation with associated ethical considerations.

## Einführung

Begünstigt durch komplexere Krankheitsbilder zumeist multimorbider und älterer Patienten ergeben sich in der modernen Intensivtherapie regelmäßig Situationen, in denen differenzialdiagnostische Überlegungen epileptische Ereignisse und somit eine antiepileptische Therapie einbeziehen. Diese sind nicht auf die Fachgebiete der Neurologie und Neurochirurgie begrenzt, da sich gerade im Falle eines Status epilepticus (SE) interdisziplinäre Schnittstellen ergeben. Der nachfolgende Beitrag fasst fachübergreifend den aktuellen Wissensstand zu antiepileptischen Therapiestrategien speziell des SE zusammen und bezieht sich ergebende intensivmedizinische Implikationen ein. Berücksichtigt wird insbesondere die im Oktober 2020 veröffentlichte Leitlinienfortschreibung der Deutschen Gesellschaft für Neurologie zum SE [60].

## Begrifflichkeiten, Epidemiologie und Therapieprinzipien

### Epileptischer Anfall

Als epileptischer Anfall werden vorübergehende klinische Symptome definiert, die mit einer abnormal gesteigerten oder synchronen neuronalen Aktivität im Gehirn einhergehen [23]. Die Symptomatik reicht vom „typischen“ epileptischen Anfall mit unwillkürlichen motorischen Entäußerungen bis hin zu stereotypen Bewegungen. Die motorischen Entäußerungen können dabei die mimische Muskulatur, die Extremitäten und den Rumpf einbeziehen. Diese treten entweder bilateral oder als einseitige Entäußerungen auf und können auch nur auf einzelne Bereiche wie beispielsweise einzelne Finger einer Hand oder Teile der mimischen Muskulatur begrenzt sein. Einseitige motorische Entäußerungen können mit einer begleitenden Bewusstseinsstörung einhergehen. Diese kann jedoch auch fehlen, wohingegen bilaterale Entäußerungen in der Regel mit einer Einschränkung des Bewusstseins assoziiert sind. Deutlich schwieriger erkennbar sind Präsentationen von epileptischen Anfällen, bei denen es zu einer alleinigen Veränderung des Bewusstseins ohne begleitende motorische Entäußerungen kommt. Die Indikation zur antiepileptischen Therapie bedarf dabei der sorgfältigen Evaluation und orientiert sich v. a. am Umstand einer behobenen oder fortbestehenden Ursache bzw. dem Risiko von Komplikationen im Falle erneut auftretender Ereignisse. Im intensivmedizinischen Kontext erscheint die Differenzierung der nachfolgenden Situationen am bedeutsamsten.

### Akut symptomatische epileptische Anfälle

Diese treten im engen zeitlichen Zusammenhang mit einer akuten Hirnschädigung auf, die metabolischer oder toxischer Genese bzw. durch eine strukturelle oder infektiöse Affektion des Gehirns, auch als Begleitsymptom eines systemischen Prozesses wie einer Infektion oder einer Hypoglykämie, begünstigt sein kann. Der zeitliche Zusammenhang richtet sich nach der zugrunde liegenden Erkrankung und ist beispielsweise bei zerebrovaskulären Ereignissen auf eine Woche beschränkt [8]. Akut symptomatische Anfälle stellen mit 45 % den größten Anteil der medikamentös behandelten epileptischen Anfälle auf Intensivstationen dar [81]. In nur 20–30 % der Fälle gehen sie mit der Ausprägung einer Epilepsie einher [30]. Eine zwingende Indikation zur antiepileptischen Therapie ergibt sich somit zum Zeitpunkt des Auftretens in der Regel nicht.

### Epilepsie

Die Diagnosestellung einer Epilepsie setzt den Nachweis einer gesteigerten Erregbarkeit des Gehirns voraus, welche abzugrenzen ist von einem häufig temporären Zustand bei akut symptomatischen Anfällen. Der Nachweis der gesteigerten Erregbarkeit wird erbracht durch das Auftreten von 2 unprovozierten epileptischen Anfällen im Abstand von mehr als 24 h oder durch entsprechende Befunde in der Zusatzdiagnostik, d. h. einen epileptogenen Fokus in der zerebralen Magnetresonanztomographie (MRT) oder, weniger geeignet, in der Computertomographie (CT) sowie typische Befunde in der Elektroenzephalographie (EEG) [60]. Die Diagnose einer Epilepsie geht in den meisten Fällen mit einer antiepileptischen Therapie einher, deren Änderung oder Absetzen nicht ohne die Einbeziehung eines in diesem Feld erfahrenen Neurologen erfolgen sollte.

### Psychogene bzw. dissoziative Anfälle

Diese stellen eine relevante, klinisch häufig schwer abzugrenzende Differenzialdiagnose gegenüber epileptischen Anfällen dar. Für psychogene Anfälle sprechen asynchrone Bewegungen der Extremitäten mit wechselnder Seitenbetonung und Intensität, das Hin‑/Herwerfen des Kopfes, ruckartige Bewegungen des Beckens und das Zusammenkneifen der Augen [3]. Jedoch ist keine dieser Beobachtungen pathognomonisch für einen psychogenen Anfall [4]. Zur diagnostischen Abgrenzung ist die simultane EEG essenziell, in der epileptische Muster typischerweise fehlen. Psychogene Anfälle führen aufgrund der diagnostischen Unsicherheit immer wieder zu aggressiven Therapiemaßnahmen bis hin zur Intubation. Die richtige Diagnosestellung war in einer Beobachtungsstudie mit einer 94%igen Reduktion der Vorstellungen in der Notaufnahme und einem vollständigen Rückgang von Hospitalisierungen assoziiert [53].

### Status epilepticus

Als SE wird ein Zustand anhaltender epileptischer Aktivität bezeichnet, welche in der Regel nicht selbstlimitierend ist. Die Sterblichkeit ist mit 20 % relativ hoch [78]. Die Inzidenz des SE wird in Deutschland mit 20/100.000 Einwohner innerhalb eines Jahres angegeben [37]. Damit ist der SE der zweithäufigste neurologische Notfall nach dem Schlaganfall. Vor allem der Status generalisierter tonisch-klonischer Anfälle bzw. der konvulsive SE (CSE) stellt ein vital bedrohliches Krankheitsbild dar, welches der sofortigen Therapie und der Behandlung auf einer Intensivstation bedarf. Theoretische Folgen einer anhaltenden epileptischen Aktivität betreffen einerseits das Gehirn selbst und reichen bis hin zum fokalen und teils generalisierten Hirnödem, wenngleich diese Komplikationen im klinischen Alltag aufgrund der in den vergangenen Jahren scheinbar adäquateren Behandlung des SE nicht mehr vorzukommen scheinen. Zudem ergibt sich durch anhaltende motorische Entäußerungen mit begleitender Hyperthermie und Rhabdomyolyse das Risiko für Organdysfunktionen auch außerhalb des Gehirns, wobei sich in erster Linie ein Nierenversagen ergeben könnte. Im intensivmedizinischen Umfeld gewinnt insbesondere der SE ohne vordergründige motorische Symptome – der nonkonvulsive Status epilepticus (NCSE) – an Bedeutung, weil er als Ursache einer jeden nicht anders erklärten Bewusstseinsstörung in Betracht kommt. Bislang liegen nur wenige Daten zur Inzidenz von epileptischen Ereignissen bei intensivpflichtigen Patienten vor; geschätzt wird ein kumulatives Risiko von 3,3 % [91]. Jedoch sind spezielle Phänomene wie der NCSE mit hoher Wahrscheinlichkeit unterdiagnostiziert [21, 42]. Die medikamentöse Therapie des SE erfolgt in der Regel aggressiv und reicht bis zum Einsatz von Anästhetika. Entsprechend des Therapieansprechens lassen sich die 4 Stufen (1) initialer SE, (2) etablierter SE, (3) refraktärer SE (RSE) und (4) superrefraktärer SE (SRSE) abgrenzen [78].

## Klinische Präsentation und Diagnostik des Status epilepticus

In der Akutphase eines SE hängen die Kenntnis einer im Vorfeld diagnostizierten Epilepsie, die Beurteilung der klinischen Präsentation und die einzuleitende Diagnostik unmittelbar zusammen. Beispielsweise ist im Falle einer klinisch korrelierenden Symptomatik mit Myoklonien der rechtsseitigen Extremitäten bei bekannter struktureller Epilepsie infolge eines linkshemisphäriellen Defektareals vor bzw. begleitend zur Therapieeinleitung die Spiegelbestimmung der antikonvulsiven Dauermedikation entscheidend, um die Prophylaxe nach behandeltem SE bestmöglich anzupassen. Die diagnostischen Verfahren, die bei SE in der initialen Differenzialdiagnostik zum Einsatz kommen, sind in der Tab. 1 zusammengefasst.

**Table 1:** 

Anamnese	Epilepsie? Hinweise für Intoxikation oder Trauma? Zeichen einer anderen Erkrankung (z. B. Fieber)? Medikamente bzw. Substanzabusus?
Klinische Untersuchung	Fokale neurologische Symptome? Meningismus?
Labordiagnostik	Blutzucker Elektrolyte (Na, Ca, Mg, K) Blutbild Blutgasanalyse Entzündungsparameter (CRP, ggf. PCT) Gerinnung Leberfunktionsparameter (ggf. auch Ammoniak) Nierenretentionsparameter Kreatinkinase Schilddrüsenhormone Toxikologisches Screening (Urin, Blut) Antiepileptikaspiegel bei behandelter Epilepsie
Mikrobiologische Diagnostik	Mediengewinnung, u. a. Lumbalpunktion
Apparative Diagnostik	EEG (v. a. bei vermutetem NCSE) cCT, niederschwellig mit Kontrastmittel cMRT

*Na* Natrium, *Ca* Kalzium, *Mg* Magnesium, *K* Kalium, *CRP* C-reaktives Protein, *PCT* Prokalzitonin, *EEG* Elektroenzephalographie, *cCT/cMRT* zerebrale CT/MRT, *NCSE* nonkonvulsiver Status epilepticus

### Klinische Präsentation

Während die konvulsiven Formen des SE in der Regel rein klinisch anhand der zumeist motorischen Entäußerungen der Extremitäten diagnostiziert werden, kann bei den Formen ohne vordergründige motorische Symptome (NCSE) anhand klinischer Kriterien zunächst nur der Verdacht auf das Vorliegen eines SE geäußert werden. Da der NCSE eine höhere Prävalenz in der älteren Bevölkerung aufweist [59], sollte dieser gerade bei älteren Patienten mit folgenden Symptomen in Betracht gezogen werden: (1) akut aufgetretener Verwirrtheitszustand bzw. akut eingesetzte kognitive Beeinträchtigung [45], (2) ungeklärte quantitative Bewusstseinsstörung, (3) subtile motorische Phänomene (Blinzeln, Myoklonien der Gesichtsmuskulatur, am Rumpf oder an Extremitäten, nystagmusartige Augenbewegungen, forcierte Blickwendung) mit begleitender qualitativer oder quantitativer Bewusstseinsstörung [20]. Die Diagnosestellung des NCSE basiert nach jetzigem Stand auf 2 Kernkriterien: (1) der akuten Veränderung des Bewusstseinszustandes, welche von einer leichtgradigen qualitativen Bewusstseinsstörung (z. B. Verwirrtheit) bis hin zum Koma reicht, und (2) einer abnormalen Aktivität im EEG [68].

### EEG-Diagnostik

**Figure 1:**
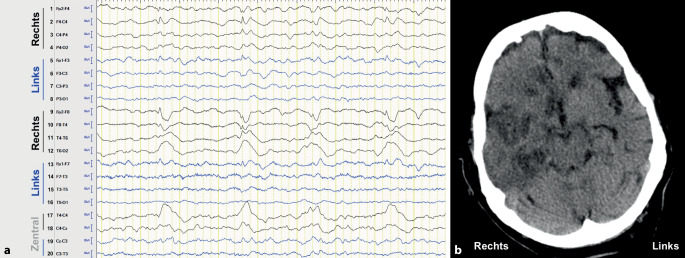

Bei konvulsiven Formen des SE ist die EEG in der Primärdiagnostik vorrangig für die Abgrenzung zum Status psychogener Anfälle sinnvoll [60]. Außerdem kommt sie zur Therapieevaluation nach klinischem Durchbrechen des konvulsiven SE, jedoch prolongierter Bewusstseinsstörung zum Einsatz, um eine anhaltende (nonkonvulsive) Anfallsaktivität nachzuweisen bzw. auszuschließen. Dagegen ist die EEG für die Diagnosestellung des NCSE in den meisten Fällen unabdingbar (Abb. 1). Klinisch kann zwischen einer kontinuierlichen und intermittierenden Anfallsaktivität zumeist nicht unterschieden werden, sodass ein statusuntypisches EEG das Vorliegen eines NCSE nicht sicher ausschließt. Die diagnostische Aussagekraft wird durch eine längere bzw. bestenfalls kontinuierliche Ableitung oder durch Wiederholungen erhöht [24, 42].

### Labordiagnostik

In der Primärdiagnostik des SE können labordiagnostisch wesentliche Ursachen für akut symptomatische epileptische Ereignisse sowie Provokationsfaktoren bei bereits diagnostizierter Epilepsie und bei der Erstmanifestation einer strukturellen Epilepsie identifiziert werden. Dazu gehören: Intoxikationen (vorrangig Alkohol, aber auch Amphetamine, Kokain, Medikamente), systemische und ZNS-Infektionen, Elektrolytentgleisungen (vornehmlich Hyponatriämie) und metabolische Störungen wie beispielsweise ein Nieren- oder Leberversagen. Dementsprechend umfangreich sollte die initiale Labordiagnostik durchgeführt werden und neben Untersuchungen aus dem Blut auch eine Diagnostik aus anderen Medien wie Urin oder Liquor umfassen [60]. Bei der Liquordiagnostik steht mit dem Nachweis eines akut-entzündlichen Syndroms zunächst die Erregerdiagnostik (bakteriell und viral) im Vordergrund. Bei diesbezüglich negativer Diagnostik und unzureichender Besserung unter antiinfektiver Therapie muss alternativ eine autoimmune Enzephalitis in Betracht gezogen werden. Dabei können Enzephalitiden infolge von Autoimmunprozessen gegen Oberflächenantigene wie beispielsweise den GABA-A/B- und NMDA-Rezeptor sowie GAD als auch gegen intrazelluläre Antigene wie Hu einen SE bedingen [26, 27, 29, 51, 65, 79].

### Bildgebende Diagnostik

Ein SE ohne vorherige Diagnose einer Epilepsie sollte stets bildgebend abgeklärt werden. In der Akutphase ist aufgrund der raschen Verfügbarkeit auch bei beatmeten Patienten meist die zerebrale CT Mittel der Wahl. Zwei bis 20 % der Patienten mit Schlaganfall entwickeln akut symptomatische epileptische Anfälle [22], sodass bei klinischem Verdacht (fokaler SE oder fokal-neurologisches Defizit) niederschwellig eine zerebrale Gefäßdarstellung ergänzt werden sollte. Auch kann die Perfusions-CT bei der Differenzierung zwischen SE und ischämischem Schlaganfall helfen, da der SE oft mit einer regionalen Hyperperfusion [46] und der ischämische Schlaganfall mit einer Hypoperfusion assoziiert sind. Fehlerbehaftet ist diese vereinfachte Interpretation jedoch in Situationen, bei denen ein spontan rekanalisierter Gefäßverschluss mit resultierender Hyperperfusion eine primär epileptische Genese vortäuscht. Bei etwa der Hälfte der Patienten mit einem SE ergibt sich zunächst keine sichere Ursachenzuschreibung, sodass sich im Verlauf eine MRT zur Fokussuche anschließen sollte [60].

## Stufengerechte Pharmakotherapie des Status epilepticus

**Figure 2:**
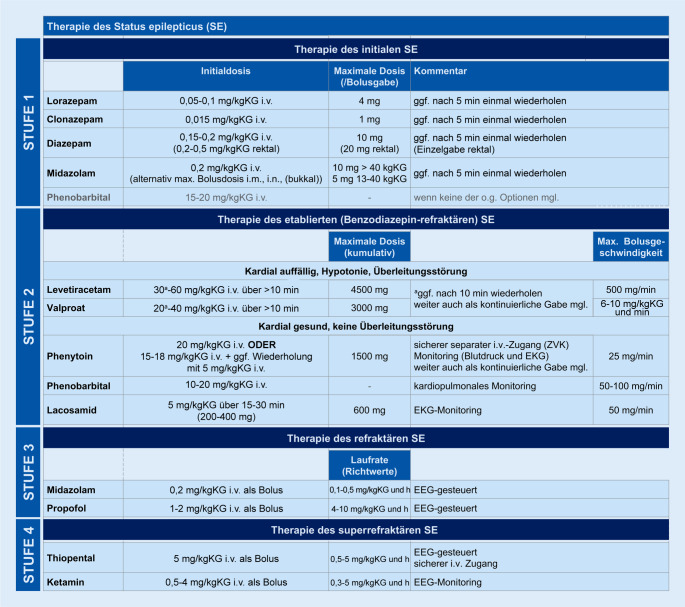

Aufgrund der Vielzahl der pharmakologischen Therapieansätze mit auf der Ebene einzelner Antiepileptika breitem Nebenwirkungsprofil sollte die Therapie des SE streng einem Stufenschema folgen (Abb. 2, in Anlehnung an [47, 60, 76]).

In Stufe 1, d. h. der Initialphase nach gerade erfolgter Diagnosestellung des SE, kommen Benzodiazepine zum Einsatz, wobei unter den Präparaten die i.v.-Anwendung von Lorazepam am besten untersucht ist. Bei fehlendem i.v.-Zugang kann Diazepam rektal oder Midazolam i.n., i.m. oder bukkal verabreicht werden. Alternative Applikationswege können erwogen werden, wenn ein verzögertes Durchbrechen eines SE infolge einer schwierigen Venenpunktion droht [2]. Wenn die Gabe von Benzodiazepinen nicht möglich ist, kann die i.v.-Phenobarbital-Gabe erwogen werden [60], welche aber aufgrund der umfangreichen Nebenwirkungen der Substanz lediglich als Reserve zu betrachten ist.

Stufe 2 beschreibt den etablierten, d. h. benzodiazepinrefraktären SE, bei dem die i.v.-Anwendung spezifischer Antiepileptika empfohlen wird, wenn der SE innerhalb der ersten 30 min nach Benzodiazepingabe in adäquater Dosierung nicht anhaltend durchbrochen ist oder die Antiepileptikagabe der Aufrechterhaltung des initialen Therapieerfolgs dienen soll. Seit der ESETT-Studie [34] werden Valproat, Levetiracetam und Fosphenytoin als gleichrangige Antiepileptika in Stufe 2 angesehen, da sich hinsichtlich der Wirksamkeit keine Unterschiede ergeben hatten. Zu beachten sind jedoch die unterschiedlich gelagerten Nebenwirkungsprofile und Kontraindikationen dieser Antiepileptika sowie die in Deutschland fehlende Verfügbarkeit von Fosphenytoin. Darüber hinaus finden Erfahrungen im Behandlerteam Berücksichtigung bei der Wahl des Antiepileptikums.

**Figure 3:**
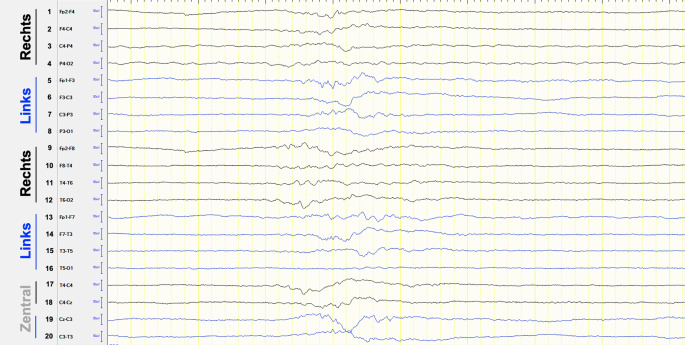

Stufe 3 bezeichnet den gegenüber der Benzodiazepin- und spezifischen Antiepileptikagabe refraktären SE (RSE). Spätestens mit diesem besteht die Indikation zur Aufnahme auf eine Intensivstation. Als Therapieeskalation stehen Anästhetika zur Verfügung, wobei die kontinuierliche i.v.-Propofol- oder i.v.-Midazolam-Gabe am geläufigsten ist. Stufe 3 wird innerhalb 1 h nach der erfolglosen Therapie mit spezifischen Antiepileptika bzw. innerhalb von 48 h nach Symptombeginn empfohlen. Die anvisierte Narkosetiefe zur Behandlung eines RSE ist bisher unklar; als Therapieziele werden die reine Anfallsunterdrückung, ein Burst-Suppression-Muster (Abb. 3) oder eine isoelektrische EEG-Kurve diskutiert [60]. Voraussetzung für eine suffiziente Therapiekontrolle ist ein kontinuierliches EEG-Monitoring, welches auch zur Abgrenzung eines aus dem CSE hervorgehenden NCSE notwendig ist [42]. Nach 12–48 h sollte sich unter fortgesetztem EEG-Monitoring ein Reduktionsversuch der Anästhetika anschließen. Treten epilepsietypische Potenziale oder gar klinische Anfallsäquivalente hierunter auf, stehen als Möglichkeiten zur Verfügung: (1) Dosiserhöhung oder Wiederbeginn des verwendeten Anästhetikums, (2) Ergänzung um ein weiteres Anästhetikum oder (3) Umstellung auf ein anderes Anästhetikum. Wenngleich sich in der Literatur keine konkreten Handlungsanweisungen finden, erscheint in der Praxis eine Kombination von (1) und (2) rational. Bei der Therapieplanung zu berücksichtigen ist die häufige Beobachtung, dass sich durch Anästhetika ein RSE in der Regel gut durchbrechen lässt, jedoch im Zuge der Reduktion oder nach deren Beendigung Rezidive v. a. dann zu erwarten sind, wenn die spezifische antiepileptische Therapie unverändert blieb. Insofern empfiehlt es sich, die Phase der Anästhetikagabe für einen Ausbau der spezifischen antiepileptischen Therapie zu nutzen, wobei die Dosiserhöhung oder Ergänzung eines Antiepileptikums oder auch die Kombination von beidem zur Anwendung kommt.

Nachfolgend werden die in der Behandlung des SE verwendeten Medikamente bzw. -gruppen vereinfacht in Anlehnung an die jeweiligen Fachinformationen und die verfügbare Übersicht von Brunton et al. [13] dargestellt.

### Benzodiazepine (Stufe 1)

Diese wirken über die Verstärkung der GABA-induzierten Hemmung im Zentralnervensystem (ZNS), indem sie durch die Bindung am GABA-A-Rezeptor die Öffnungsfrequenz der GABA-modulierten Chloridkanäle erhöhen. Nach i.v.-Gabe verhalten sich Benzodiazepine wie andere lipidlösliche Medikamente, d. h., sie gelangen schnell ins ZNS, jedoch nimmt deren Wirkung durch die Umverteilung in der Peripherie prompt wieder ab. Die Lipidlöslichkeit hängt mit der Plasmaproteinbindung zusammen, welche bei Lorazepam und Clonazepam mit 80–90 % niedriger als bei Diazepam oder Midazolam mit 95–99 % ist. Benzodiazepine werden in der Leber unter Beteiligung der Zytochrom-P450-Enzyme (CYP) metabolisiert, wodurch sich ein Interaktionspotenzial mit Enzyminduktoren und -hemmern ergibt. Zu den relevantesten Nebenwirkungen der Benzodiazepine gehören neben der Atemdepression die Hypotension und die Toleranzentwicklung.

### Spezifische Antiepileptika (Stufe 2)

Unter den spezifischen Antiepileptika werden klassische und neuere Präparate subsumiert, wobei sich Letztere durch eine bessere Verträglichkeit und eine größere therapeutische Breite sowie ein geringeres Interaktionspotenzial auszeichnen, wenngleich die Frage nach dem besten Antiepileptikum bei der SE-Behandlung unbeantwortet bleibt [11, 18, 56, 84, 85]. Verkomplizierend liegen oft multiple Wirkmechanismen vor, die teils dosisabhängig sind [39]. Speziell im intensivmedizinischen Bereich sollte ein ideales Antiepileptikum folgende Eigenschaften besitzen: (1) Möglichkeit der i.v.-Gabe, (2) rascher Wirkungseintritt, (3) gute Steuerbarkeit, (4) kein relevanter Metabolismus, (5) keine Interaktionen, (6) kein sedierender Effekt und (7) keine Toxizität. Wenngleich die Kombination aller Faktoren unerreichbar scheint, rücken die i.v.-Darreichungsformen der neueren Antiepileptika diesem Ziel zunehmend näher [9, 62]. Nachfolgend werden die wichtigsten Charakteristika der in der SE-Behandlung eingesetzten spezifischen Antiepileptika zusammengefasst:

Levetiracetam war das erste Antiepileptikum der neueren Generation, das für die i.v.-Gabe zur Verfügung stand [36, 62]. Der Wirkmechanismus ist noch immer nicht vollständig geklärt; vermutet werden Interaktionen am synaptischen Vesikelprotein (SV2A), das an der Glutamat- und GABA-Freisetzung beteiligt ist, und an den N‑Typ-Kalziumkanälen [13, 39]. In der Therapie des etablierten SE sind die Wirksamkeit und Sicherheit von Levetiracetam vergleichbar mit denen anderer empfohlener Antiepileptika [11, 85]; eine formale Zulassung für den SE liegt allerdings nicht vor. Die Halbwertszeit (HWZ) beträgt 6–8 h; Serumspiegelbestimmungen können durchgeführt werden, um eine Akkumulation zu vermeiden, allerdings bestehen Unsicherheiten zum Zielbereich (6–20 µg/ml vorgeschlagen in [50]). Die wesentlichen Vorteile von Levetiracetam bestehen in der fehlenden hepatischen Metabolisierung und der fehlenden Interaktion mit anderen Medikamenten [36, 47]. Jedoch ist eine Dosisreduktion bei Einschränkungen der Nierenfunktion notwendig; gleichzeitig ist bei terminaler Niereninsuffizienz mit intermittierenden Dialysen (IHD) eine Elimination von 50 % zu erwarten, sodass nach der IHD eine zusätzliche Gabe von 30–50 % der Tagesdosis empfohlen wird [47, 50]. Über den Einsatz bei kontinuierlichen Nierenersatzverfahren ist wenig bekannt, wobei die Standarddosierung sicher zu sein scheint [69, 80]. Zu den bedeutsamsten Nebenwirkungen zählen psychische und zentralnervöse Phänomene wie Agitation, erhöhte Reizbarkeit, Somnolenz und Kopfschmerzen, selten auch eine Thrombozytopenie.

Brivaracetam entspricht einem Abkömmling des Levetiracetams und ist in i.v.-Darreichungsform vorhanden. Es dient ebenfalls als Ligand für das SV2A mit jedoch 15- bis 30-mal höherer Affinität als sein Vorgänger und hemmt zusätzlich spannungsabhängige Natriumkanäle [13, 35]. Aufgrund der hohen Lipidlöslichkeit resultiert im Vergleich zum Levetiracetam eine bessere Penetration der Blut-Hirn-Schranke mit rascher verfügbaren Wirkspiegeln im ZNS [48]. Eine formale Zulassung für die Behandlung des SE liegt nicht vor. Retrospektive Daten weisen auf eine Effektivität bis zu 57 % hin [1, 32, 64, 72]. Die eingesetzten Tagesdosen reichten von 50–400 mg [12]. Die Metabolisierung von Brivaracetam erfolgt hepatisch unter Einbeziehung von CYP-Enzymen, und die Eliminierung erfolgt größtenteils renal. Einschränkungen der Leberfunktion sollten daher zu einer Dosisreduktion bis zu 30–50 % führen, wohingegen leichte Einschränkungen der Nierenfunktion keiner Dosisanpassung bedürfen [35, 66, 71].

Valproat ist das zuletzt entwickelte klassische Antiepileptikum in der Therapie des SE. Bekannt sind diverse Wirkmechanismen u. a. mit Interaktionen an spannungsabhängigen Natrium- und T‑Typ-Kalziumkanälen sowie im GABA-Metabolismus. Valproat ist wirksam sowohl bei generalisierten als auch fokalen Anfällen (konvulsiv und nonkonvulsiv) und ist immerhin eingeschränkt zur Therapie bei SE zugelassen [60, 77]. Die HWZ beträgt 12–16 h. Serumspiegelkontrollen sind sinnvoll, um Überdosierungen zu vermeiden, da bei Konzentrationen von mehr als 100 µg/ml vermehrt Nebenwirkungen auftreten. Valproat wird zu 95 % hepatisch metabolisiert, v. a. unter Einbeziehung der Uridin-5’-diphospho-Glucuronosyltransferase (UDP-Glucuronosyltransferase, UGT) und in kleinerem Ausmaß der CYP-Enzyme (2C9 und 2C19), und nur zu 5 % renal eliminiert. Valproat ist ein Enzyminduktor, u. a. von UGT und CYP2C9. Nebenwirkungen ergeben sich v. a. aus der Leber- und Pankreastoxizität, die von passager asymptomatischen Leberenzymerhöhungen bis zur fulminanten Hepatitis und Pankreatitis reicht. Beschrieben wurde zudem eine Hyperammonämie ohne Leberversagen, deren Entstehungsmechanismus sowie klinische Relevanz bisher unklar sind [43, 67]. Im klinischen Alltag relevant ist ferner die als Nebenwirkung bekannte Thrombozytopenie, die besonders bei intrakraniellen Blutungen der Nutzen-Risiko-Abwägung bedarf [47]. Als Kontraindikationen gelten u. a. relevante Lebererkrankungen, eine Pankreasfunktionsstörung, eine bekannte mitochondriale Erkrankung sowie eine Schwangerschaft aufgrund der teratogenen Wirkung von Valproat.

Phenytoin ist ein klassisches, in den 1930er-Jahren entdecktes Antiepileptikum, das spannungsabhängige Natriumkanäle hemmt und zur Behandlung des SE zugelassen ist [60]. Aufgrund der schlechten Wasserlöslichkeit beinhaltet die i.v.-Phenytoin-Lösung die Substanzen Propylenglykol und Ethanol; durch den resultierenden stark alkalischen pH können schwere lokale Hautreaktionen v. a. bei Para- bzw. Extravasation auftreten, bis hin zum „purple glove syndrome“. Außerdem bilden sich mit manchen Injektionslösungen (z. B. Glucose) Präzipitate, sodass die Substanz in der Regel unverdünnt angewendet wird. Phenytoin sollte über einen sicheren und separaten venösen Zugang in einer großkalibrigen Vene bzw. vorzugsweise über einen zentralen Venenkatheter injiziert werden. Empfohlen wird zudem eine langsame Injektion mit entsprechend den Herstellerangaben nicht mehr als 25 mg Phenytoin/min. Beim Einsatz von Phenytoin ist auch das hohe, teils schwer abschätzbare Interaktionspotenzial zu beachten, welches auf einer hohen Proteinbindung (95 %), einer nonlinearen Eliminationskinetik mit vom Plasmaspiegel abhängiger HWZ sowie dem vorwiegend hepatischen Metabolismus unter Einbeziehung u. a. der Enzyme CYP2C9 und CYP2C19 beruht. Phenytoin bedingt eine starke Enzyminduktion der UGT- und CYP-Systeme und gleichzeitig eine Inhibition von CYP2C9. Serumspiegelkontrollen sind sinnvoll, um toxische Dosierungen (Konzentrationen von mehr als 25 µg/ml) frühzeitig zu erkennen. Durch den großen HWZ-Bereich von 20–60 h gehen Dosisänderungen nur prolongiert mit einer Änderung der Serumkonzentration einher, was bei der Therapiesteuerung bedacht werden sollte. Aufgrund kardialer Nebenwirkungen (atriale und ventrikuläre Reizleitungsstörungen, Hypotension) ist das Monitoring von EKG und Blutdruck obligat. Relevant sind zudem Nebenwirkungen im Bereich des ZNS wie Ataxien und Nystagmen. Zu den Kontraindikationen gehören eine schwere Herzinsuffizienz und kardiale Reizleitungsstörungen, v. a. Bradykardien und Blockbilder sowie eine Hypotonie und eingeschränkte Leberfunktion.

Phenobarbital gehört zu der Gruppe der Barbiturate, dessen Einsatz bis in das Jahr 1912 zurückreicht [39, 56]. Der Wirkmechanismus beinhaltet eine Modulation am GABA-A-Rezeptor [28], woraus die Verstärkung der GABA-Affinität zum Rezeptor und damit eine erhöhte Krampfschwelle durch eine Membranhyperpolarisation resultieren. Zusätzlich unterdrückt Phenobarbital die präsynaptische Freisetzung von exzitatorischen Neurotransmittern. Anders als die meisten Barbiturate (z. B. Thiopental, s. unten) besitzt Phenobarbital die Eigenschaft, in niedrigeren Dosierungen epileptische Potenziale zu unterdrücken, ohne einen relevant sedierenden Effekt hervorzurufen. Die HWZ ist mit 75–120 h relativ lang, was bei der Therapiesteuerung bedacht werden sollte. Serumspiegelbestimmungen sind sinnvoll, wobei toxische Konzentrationen ab 50 µg/ml erreicht werden. Die Metabolisierung erfolgt vorwiegend hepatisch unter Einbeziehung u. a. von CYP2C9 und UGT, was in Anbetracht der gleichzeitig starken Induktion beider Enzymsysteme zu Interaktionen führt. Bis zu 40 % werden pH-abhängig renal eliminiert. Beim Einsatz von Phenobarbital ist ein kontinuierliches Monitoring aufgrund der Gefahr der kardiorespiratorischen Depression obligat. Als weitere Nebenwirkungen wurden schwere Hautreaktionen bis zum Steven-Johnson-Syndrom beschrieben. Als Kontraindikationen gelten schwere Lebererkrankungen; zu beachten sind zudem schwere Nieren- oder Herzinsuffizienz.

Lacosamid ist eine funktionalisierte Aminosäure (L-Serin); als deren Wirkmechanismus wird die Verstärkung der langsamen Inaktivierung der spannungsabhängigen Natriumkanäle angenommen [13, 39]. Die Anwendung beim SE ergibt sich v. a. aus der i.v.-Darreichungsform und aus ersten vergleichenden Untersuchungen mit hoffnungsvollen Ergebnissen, wenngleich für den SE keine Zulassung vorliegt [47, 60]. Die HWZ ist mit 12–16 h vergleichsweise kurz. Therapeutische Serumspiegel werden im Bereich von 5–20 µg/ml vermutet. Der Metabolismus ist weitgehend unklar; ein Abbau über CYP-Enzyme (3A4, 2C9, 2C19) wird vermutet, und die Elimination erfolgt zu 95 % renal. Die hauptsächlich renale Elimination bedingt eine Dosisanpassung bei vorhandener Niereninsuffizienz. Die Dosierungsempfehlungen bei IHD beziehen sich auf ambulante Patienten mit terminaler Niereninsuffizienz. Für den Einsatz während eines kontinuierlichen Nierenersatzverfahrens existieren kaum Daten. Daher sind individuelle Anpassungen der Lacosamiddosierung unter Berücksichtigung der Serumkonzentration notwendig [25, 82]. Ein engmaschiges Monitoring der Herzfunktion wird insbesondere bei höheren Dosierungen und gleichzeitiger Therapie mit anderen PR-Intervall-verlängernden Medikamenten empfohlen [47]. Als Kontraindikationen gelten AV-Blockierungen II° und III°.

### Interaktionen spezifischer Antiepileptika

Bei der Anwendung ergeben sich zahlreiche Interaktionen zwischen den Antiepileptika selbst, aber auch gegenüber anderen Medikamenten, von denen nachfolgend die mit der vermutlich größten klinischen Bedeutsamkeit dargestellt werden. Da diese Aufstellung keinen Anspruch auf Vollständigkeit hat, sind im individuellen Fall Interaktionen immer zu bedenken und niederschwellig pharmakologische Beratungen wahrzunehmen.

Durch die hohe Proteinbindung einiger Antiepileptika (z. B. Valproat und Phenytoin) ergeben sich in Situationen mit verminderter Eiweiß‑/Albuminkonzentration im Serum, wie sie beispielsweise bei Nieren- oder Lebererkrankungen sowie im längeren intensivmedizinischen Behandlungsverlauf auftreten, höhere Konzentrationen der freien und somit klinisch wirksamen Fraktion der Antiepileptika. Dabei können Intoxikationen bei gleichzeitig noch normaler Serumkonzentration des Medikaments entstehen. Der Effekt kann potenziert werden, wenn beispielsweise Phenytoin durch eine Valproatkomedikation aus Proteinbindungsstellen verdrängt und zusätzlich der Abbau gehemmt wird.

Auf der Ebene der hepatischen Metabolisierung ergeben sich teils schwer vorherzusehende Effekte. Meist resultieren Interaktionen aus der Enzyminduktion oder -inhibition, wobei die beiden Leberenzymsysteme CYP und UGT die größte Bedeutung einnehmen [39, 86]. Beispielsweise können Enzyminduktoren den Abbau von Valproat beschleunigen, wobei Valproat selbst als Enzyminhibitor den Metabolismus von Phenytoin und Phenobarbital relevant hemmen kann [86]. Eine Überwachung der Serumspiegel bzw. der freien Medikamentenfraktion kann bei der Einschätzung helfen. Das CYP-System ist bekanntermaßen auch verantwortlich für den Metabolismus von vielen anderen Medikamenten, deren Stoffwechsel durch die gleichzeitige Gabe von Enzyminduktoren gesteigert wird. Barbiturate bedingen somit einen erhöhten Metabolismus von Warfarin, was eine Dosiserhöhung von diesem bis zum Zehnfachen der Ausgangsdosis notwendig macht. Besonders gefährlich ist diese Interaktion im Falle des Absetzens des Enzyminduktors, wodurch sehr hohe Spiegel von Warfarin/Phenprocoumon mit Blutungskomplikationen entstehen. Die Wechselwirkung zwischen Phenytoin und Warfarin ist deutlich komplexer und kann sowohl eine Erhöhung als auch Verminderung der Warfarinkonzentration zur Folge haben. Valproat erhöht das Risiko von Blutungskomplikationen bei gleichzeitiger Therapie mit Warfarin/Phenprocoumon nicht nur durch eine Enzyminhibition, sondern auch durch eine direkte Beeinträchtigung der Blutgerinnung.

Eine weitere intensivmedizinisch relevante Interaktion besteht zwischen den Enzyminduktoren und den nach Leber‑, Nieren- und Herztransplantation eingesetzten Immunsuppressiva Tacrolimus und Cyclosporin A, woraus verminderte Spiegel resultieren, bis hin zur Organabstoßung.

Eine relevante Interaktion besteht zudem zwischen Valproat und Carbapenemantibiotika. Durch einen bisher unklaren Mechanismus kommt es zur starken Reduktion des Valproatspiegels [7, 83]. Eine Dosiseskalation von Valproat führt in den meisten Fällen nicht zu einem suffizienten Spiegelaufbau, sodass im klinischen Alltag meist ein alternatives Präparat für die Dauer der Antibiotikatherapie eingesetzt wird.

### Anästhetika (Stufe 3)

Der Wirkmechanismus von Anästhetika, v. a. im Rahmen der SE-Behandlung, ist nicht geklärt. Vermutet werden vielfältige Modulationen von GABA-A- und glycinrezeptorgesteuerten Chloridkanälen sowie glutamatgesteuerten NMDA-Rezeptoren, die in Verbindung mit Kalziumströmen stehen. Die klinische Wirkung von parenteral applizierten Anästhetika hängt eng mit deren Lipophilie zusammen, die innerhalb kürzester Zeit zu einem suffizienten Wirkspiegel im Gehirn führt. Parenteral verabreichte Anästhetika besitzen eine kontextsensitive HWZ in Abhängigkeit vom Stoffwechsel und von der Lipophilie des eingesetzten Präparates, sodass eine verlängerte Elimination nach wiederholter bzw. kontinuierlicher Gabe resultiert. Die HWZ verlängert sich bei der kontinuierlichen Gabe von Midazolam, Propofol oder Ketamin nur moderat, wohingegen die kontinuierliche Applikation von Thiopental eine ausgeprägte Verlängerung der HWZ nach sich zieht [13]. Vor dem Einsatz der Anästhetika muss eine kardiorespiratorische Überwachung etabliert sein, und Mittel zur Narkoseeinleitung wie kreislaufunterstützende Medikamente und das Intubationsinstrumentarium müssen vorbereitet vorliegen. Im Detail werden verwendet (Dosierungen in Abb. 2):

Midazolam aus der Gruppe der Benzodiazepine wurde bereits in Stufe 1 referiert. Midazolam besitzt eine vergleichsweise kurze HWZ, kann deswegen auch kontinuierlich eingesetzt werden, mit relativ großer Dosierungsbreite.

Propofol ist unter Raumtemperatur ein Öl. Die i.v.-Emulsion enthält Sojaöl und Eilecithin, was allergische Reaktionen auslösen kann, z. B. bei Patienten mit Erdnuss‑/Sojaallergie, obgleich der Zusammenhang zwischen Nahrungsmittel- und Propofolallergie fraglich ist [70]. Bei der kontinuierlichen Gabe sollte Propofol als Lipidlösung bei der Gesamtlipidzufuhr berücksichtigt werden, gerade bei erhöhter Lipidzufuhr wie der ketogenen Diät. Propofol führt in den meisten Fällen zuverlässig zum Burst-Suppression-Muster in der EEG; Voraussetzung ist eine ausreichende Dosierung, die sich am besten direkt an der EEG-Ableitung orientiert. Es senkt den Sauerstoffverbrauch im Gehirn, den zerebralen Blutfluss sowie den intrakraniellen und intraokulären Druck. Metabolisiert wird Propofol zu einem weniger aktiven Produkt, das renal eliminiert wird. Neben einer allgemeinen Kreislaufdepression gilt das seltene, aber potenziell letale Propofolinfusionssyndrom (PRIS) als die bedeutsamste Nebenwirkung. Als pathophysiologische Grundlage werden eine Störung der mitochondrialen Funktion und eine toxische Lipidakkumulation vermutet [38]. Ein PRIS kann sich in Form der folgenden Symptome manifestieren: metabolische (Laktat)Azidose, Rhabdomyolyse, Fieber, Hyperlipidämie und Lebervergrößerung, bis hin zum Herz-Kreislauf- und Nierenversagen mit Notwendigkeit entsprechender Organersatz- und Unterstützungssysteme [33, 38]. Das Auftreten eines PRIS wird mit höheren Propofoldosen (> 4 mg/kgKG und h) und einem längeren Therapiezeitraum (> 48 h) in Verbindung gebracht. Diese beiden Faktoren und die Kontraindikationen entsprechend den Herstellerangaben zur Sedierung im Rahmen der Intensivtherapie sollten daher bei der Anwendung unbedingt Berücksichtigung finden. Als potenziell reversible Nebenwirkung kommt der Früherkennung und einer Beendigung der Propofolgabe bereits im Verdachtsfall eine entscheidende Bedeutung zu. Mithin gehören engmaschige Laborkontrollen beispielweise hinsichtlich der Entwicklung einer Acidose oder einer Rhabdomyolyse, wie sie durch einen Anstieg der Kreatinkinase und des Myoglobins erkennbar wird, zu sinnvollen Maßnahmen.

## Therapie des superrefraktären Status epilepticus

Bleiben die vorgenannten Therapieelemente erfolglos oder kommt es hierunter zu Anfallsrezidiven, liegt ein SRSE vor. Die zur weiteren Behandlung infrage kommenden, teils adjuvanten medikamentösen und nichtmedikamentösen Ansätze werden auch als Stufe 4 (Abb. 2) der SE-Behandlung angesehen und zeichnen sich v. a. dadurch aus, dass für diese keine bzw. eine nur sehr geringe Evidenz vorliegt [13, 60].

### Barbiturate

In 2 retrospektiven Studien wurde die hochdosierte i.v.-Gabe der Barbiturate Pentobarbital [52] und Phenobarbital [14] über eine mittlere Dauer von 6 bzw. maximal 25 Tagen untersucht. Nach dem vorangegangenen Therapieversagen von Midazolam und Propofol gelang eine Anfallskontrolle mit Raten bis zu 50 %. Als Ultima-Ratio-Ansatz beim SRSE können Barbiturate daher erwogen werden, wobei in Deutschland lediglich Thiopental zur hochdosierten kontinuierlichen Gabe zur Verfügung steht [60]. Die Wirkung von Thiopental tritt schnell ein und hält nach der Einmalgabe nur kurz an (Wirkdauer von 5–15 min). Der langsame Stoffwechsel und ein großes Verteilungsvolumen führen jedoch zu einer relevanten Kumulation und einer deutlich prolongierten und bis zu Tagen andauernden Bewusstseinsstörung nach einer kontinuierlichen Gabe. Hieraus resultiert im Vergleich zum Propofol meist eine längere mechanische Ventilationsdauer [61]. Die Metabolisierung verläuft hepatisch mit sowohl aktiven als auch inaktiven Metaboliten mit letztlich renaler Elimination. Bei der Therapieplanung zu berücksichtigen ist die Induktion von CYP-Enzymen [63]. Unter den Nebenwirkungen sind v. a. die starke kardiorespiratorische Depressivität sowie ein immunsupprimierender und histaminfreisetzender Effekt zu beachten.

### Ketamin

Das Racemat aus R+ und S‑Stereoisomeren wirkt als potenter NMDA-Antagonist. Erfahrungen zum Einsatz von Ketamin beim schwer zu behandelnden SE basieren lediglich auf Fallberichten [57]. Dennoch herrscht Konsens, dass Ketamin in der Behandlung des SRSE erwogen werden kann [60]. In der EEG resultiert eine diffuse Verlangsamung bzw. diffuse β‑Aktivität, was möglichweise dem Burst-Suppression-Muster gleichzusetzen ist. Ketamin löst einen kataleptischen Zustand aus, auch dissoziative Anästhesie genannt, bei dem das Bewusstsein und die Spontanatmung ebenso wie Muskeltonus und Schutzreflexe erhalten sein können, allerdings nicht die Willkürmotorik und das Aufforderungsbefolgen. Die Metabolisierung erfolgt hepatisch unter Einbeziehung von CYP-Enzymen (v. a. 3A4), was Interaktionen zur Folge haben kann. Durch einen sympathikomimetischen Effekt geht Ketamin in der Regel nicht mit einer kardialen Depression einher, wodurch eine iatrogen bedingte Katecholamintherapie potenziell vermieden werden kann. Zu den wesentlichen Nebenwirkungen gehören neben psychischen Phänomenen (u. a. Albträume) ein Anstieg des Blutdrucks und des intrakraniellen Drucks sowie kardiale Arrhythmien.

### Lidocain und Allopregnanolon

Eine Übersichtsarbeit von Zeiler et al. fasst bisherige Erfahrungen der Lidocaingabe mit unterschiedlichen Dosierungen und Applikationsformen bei verschiedenen Stadien des SE und variierenden Vortherapien zusammen und berichtet über ein Ansprechen von mehr als 60 % [88]. Nachdem Lidocain in der früheren Leitlinie zur Behandlung des SE auf der Grundlage kasuistischer Berichte erwähnt wurde [58], enthält die aktuelle Leitlinienfortschreibung keine Informationen mehr zum Lidocain [60], sodass die Gabe nicht empfohlen werden kann. Die Anwendung des Neurosteroids Allopregnanolon, welches die Aktivität des GABA-A-Rezeptors modulieren kann und somit hypothetisch positive Effekte auf die Entstehung epileptischer Anfälle aufweist [41], wird aufgrund der fehlenden klinischen Evidenz bisher nicht empfohlen [60].

### Klassische (enteral zu verabreichende) Antiepileptika

Für den AMPA-Rezeptor-Antagonisten Perampanel wird eine synergistische Wirkung bei begleitender Gabe von Benzodiazepinen bei der Behandlung des SE angenommen [31]. In einer deutschen Studie wurde Perampanel nach dem Versagen von insgesamt 5 antikonvulsiven Medikamenten eingesetzt und erreichte ein Ansprechen bei immerhin 4 von 10 Patienten [54]. Für Topiramat sind verschiedene antikonvulsive Wirkmechanismen bekannt, die auch eine antagonistische Wirkung am AMPA-Rezeptor einschließen. Eine Übersichtsarbeit von Brigo et al. fasst die klinischen Daten zusammen und beinhaltet auch 6 Patienten mit einem SRSE, bei denen Topiramat als letztes Medikament eingesetzt wurde und mit einem Ende der Anfallsaktivität einherging [10]. Der Einsatz genannter klassischer Antiepileptika kann somit beim SRSE erwogen werden, wenngleich mit einem Effekt zumeist erst nach Tagen bis Wochen zu rechnen ist [60].

### Volatile Anästhetika

Als Ultima-Ratio-Ansatz in der Behandlung des SRSE kann der Einsatz volatiler Anästhetika erwogen werden [60]. In einer Übersichtsarbeit von Zeiler et al. werden zwar Raten von durchschnittlich 93 % für das Erreichen eines Burst-Suppression-Musters und damit ein Durchbrechen des SE beschrieben, allerdings traten nach Beendigung der Therapie wieder epilepsietypische Potenziale auf, sodass es sich entsprechend dem aktuellen Wissensstand nicht um einen nachhaltigen Ansatz handelt [90]. Am häufigsten wurde Isofluran (Dosierungen 0,5–1,0 Vol.-%, in Einzelfällen sogar bis 3–5 Vol.-%), aber auch Desfluran (1–4 Vol.-%) sowie einmalig Xenon (30 Vol.-%) verwendet. Vorteile volatiler Anästhetika könnten sich aus der guten Steuerbarkeit ergeben, durch die eine raschere Therapieeskalation gegenüber anderen Ultima-Ratio-Ansätzen erzielbar wäre. Limitationen in der Anwendung ergeben sich aktuell v. a. aufgrund der notwendigerweise technischen Voraussetzungen, die sowohl die kontrollierte Einleitung volatiler Anästhetika als auch deren Elimination betreffen.

### Ketogene Diät

Ziel dieser ist die Bildung von Ketonkörpern infolge einer kohlenhydratarmen Diät mit resultierender Zunahme mehrfach ungesättigter Fettsäuren. Wenngleich für Ketonkörper, welche die Blut-Hirn-Schranke passieren können, eine antiepileptische Wirkung angenommen wird, ist der exakte Wirkmechanismus der ketogenen Diät nicht geklärt [55]. Infolge einer vielversprechenden retrospektiven Fallserie zum Einsatz der ketogenen Diät beim SRSE [75] wurde eine multizentrische Phase-1/2-Studie durchgeführt, in welcher bei 11 von 14 Patienten ein Sistieren der Anfallsaktivität erreicht werden konnte [16]. Als wesentliche Nebenwirkungen wurden metabolische Acidose, Hypoglykämie und Hyponatriämie berichtet. Die ketogene Diät gilt als praktikabel und sicher, sodass diese unter Nutzen-Risiko-Abwägung beim SRSE empfohlen wird [60].

### Epilepsiechirurgie und Vagusnervstimulation

Ebenfalls für den schwer zu therapierenden SE können im Einzelfall chirurgische Eingriffe erwogen werden [60]. In einer vergleichsweise kleinen Fallserie waren positive Effekte eines epilepsiechirurgischen Eingriffs nachvollziehbar [6]. Voraussetzung für die hochselektive Entfernung von Hirngewebe ist jedoch die Identifikation eines umschriebenen epileptogenen Fokus [31]. Positive Effekte wurden auch für die Vagusnervstimulation beim SRSE beschrieben [19], wenngleich eine Empfehlungsstärke aufgrund des Publikation-Bias aktuell offen bleiben muss [60].

### Elektrokonvulsive Therapie

Für diese existieren positive Berichte aus lediglich kasuistischen Arbeiten [87], sodass eine Empfehlungsstärke bislang nicht formuliert werden konnte, die Anwendung im Einzelfall beim SRSE jedoch in Erwägung gezogen werden kann [60].

### Temperaturmanagement

Analog zu akuttherapeutischen Überlegungen in anderen Gebieten wie dem Herz-Kreislauf-Stillstand und dem malignen Hirninfarkt wurde die Hypothese positiver Effekte einer Hypothermie auch beim SE formuliert [89]. Jedoch konnte die im Jahr 2016 veröffentliche HYBERNATUS-Studie mit einem prospektiven randomisierten Design unter Einschluss von 268 Patienten mit einem CSE keinen Vorteil für eine Hypothermie (32–34 °C für 24 h) gegenüber der Standardbehandlung in Bezug auf das Outcome nach 3 Monaten zeigen [40]. Zudem wurden in der Hypothermiegruppe mehr unerwünschte Nebenwirkungen, hier vorrangig Pneumonien, beobachtet. Als Therapieempfehlung ergibt sich derzeit das Einhalten einer Normothermie mit konsequenter Behandlung von Temperaturen von mehr als 37,5 °C [60].

## Intensivmedizinische Implikationen

Das im intensivmedizinischen Sprachgebrauch oft verwendete, im Detail jedoch vielschichtige und zumeist ressourcenintensive Prinzip der allgemeinen Homöostase nimmt beim SE eine zentrale Rolle ein. Abweichungen beispielweise in Form einer Hypoxämie, einer Elektrolytstörung, eines Infektgeschehens oder einer metabolischen Störung können Trigger sowohl für den Beginn als auch die Aufrechterhaltung des SE sein. Somit sollen diese Faktoren engmaschig reevaluiert und niederschwellig behandelt werden, was sich in der aktuellen Leitlinienfortschreibung beispielweise in Form einer Glucosegabe bereits im Verdachtsfall einer Hypoglykämie, der Thiamingabe gerade beim ethanolassoziierten SE und dem Ziel der suffizienten Oxygenierung (mind. 95 %ige periphere Sauerstoffsättigung) sowie der permanent vorhandenen Intubationsbereitschaft widerspiegelt [60].

Darüber hinaus stellt der therapierefraktäre SE für Intensivmediziner therapeutisch wie auch ethisch eine Herausforderung dar. Neben offenen Fragen wie der optimalen Pharmakokinetik i.v. zu applizierender Antiepileptika (kontinuierliche vs. diskontinuierliche Gabe), betrifft eine aktuelle Kontroverse die adäquate Behandlung des NCSE, für den zum jetzigen Zeitpunkt keine einheitlichen Therapieempfehlungen vorliegen. Das Themenfeld umfasst die lange Zeit umstrittene Pathogenität des Krankheitsbildes, da unmittelbar lebensbedrohliche Auswirkungen wie beispielsweise eine Laktatazidose infolge von Konvulsionen oder eine respiratorische Insuffizienz oft fehlen [17], sodass ein aggressives, potenziell komplikationsbehaftetes Therapieregime diskutiert werden muss. Andererseits konnte inzwischen das Vorliegen eines Komas im Zusammengang mit dem NCSE als Prädiktor für eine schlechte Prognose identifiziert werden [74], sodass ein konsequentes therapeutisches Vorgehen wiederum nachvollziehbar scheint. Insbesondere beim NCSE ist die Eskalation unter Einbezug der Anästhetika und damit einhergehend der Intubation ein vielfältig diskutierter Therapieschritt, der gerade bei multimorbiden bzw. älteren Patienten ethische Aspekte einbezieht [60]. Neuere Daten stützen zwar den frühen Einsatz von Anästhetika beim therapierefraktären SE [44], jedoch müssen potenzielle Komplikationen und Folgen der Atemwegssicherung [49] und einer tiefen Sedierung wie ein erhöhtes Infektionsrisiko, eine erhöhte Mortalität und eine allgemein schlechtere Langzeitprognose berücksichtigt werden [5, 15, 73]. Zur Therapieplanung ist daher die individuelle Therapiezieldefinition auf der Basis patienteneigener Wertevorstellungen notwendig, wofür ein intensiver Austausch mit Angehörigen unerlässlich ist.

## Fazit für die Praxis

- Die Diagnostik und Therapie epileptischer Ereignisse und speziell des Status epilepticus (SE) bleibt eine Herausforderung, da gerade bei Formen mit vorwiegend nichtmotorischen Symptomen klinische und elektroenzephalographische Expertise erforderlich ist.
- Die im Jahr 2020 erfolgte Fortschreibung der Leitlinie zur SE-Behandlung hält an der streng stufengerechten Therapie unter eskalierender Anwendung von Benzodiazepinen, spezifischen Antiepileptika und Anästhetika fest.
- Eine umfassende Kenntnis über die Vielzahl der zur Verfügung stehenden Substanzen mit antiepileptischer Wirkung ist notwendig, um die für den einzelnen Patienten bestmögliche Therapie zu gewährleisten.
- Gerade bei therapierefraktären Formen des SE ergeben sich intensivmedizinische Implikationen, die der interdisziplinären Schnittstellenbildung bedürfen.
